# Supplementary material for: Funding and policy incentives to encourage implementation of point-of-care C-reactive protein testing for lower respiratory tract infection in NHS primary care: a mixed-methods evaluation
Source: BMJ Open. 2018 Oct 25;8(10):e024558. doi: 10.1136/bmjopen-2018-024558 (PMC6224752; doi:10.1136/bmjopen-2018-024558)
Supplement: Supplementary file 1 [file bmjopen-2018-024558supp001.pdf]

Appendix A: Primary care testing in the NHS health system

In the UK healthcare is free at the point of contact and funded through central taxation. There is no co-payment or health insurance needed for patients to access the NHS. General practices rarely perform tests on the primary care site, with few exceptions such as urine dip tests, pregnancy tests and sometimes coagulation monitoring. The majority of laboratory tests are organised by the local hospital, with patients either attending the hospital directly for sampling, or samples being taken at the general practice and sent to the laboratory. Payment for the test is by way of a 'block contract' arrangement, and paid for by the CCG so that neither the patient nor the practice bear any cost. Under the current contractual arrangement the general practice is unable to charge the patient for NPTs and so, if performed, bears the full cost from its own income, as well as an additional time burden. For these reasons NPTs such as CRP are unfamiliar to practitioners in the UK, necessitating an examination of alternative means to encourage their implementation.
