# Supplementary material for: Funding and policy incentives to encourage implementation of point-of-care C-reactive protein testing for lower respiratory tract infection in NHS primary care: a mixed-methods evaluation
Source: BMJ Open. 2018 Oct 25;8(10):e024558. doi: 10.1136/bmjopen-2018-024558 (PMC6224752; doi:10.1136/bmjopen-2018-024558)
Supplement: Supplementary file 2 [file bmjopen-2018-024558supp002.pdf]

Appendix B: Results from Herts Valleys CCG implementation study

Five intervention practices with a total list size of 63,743 patients recorded 682 eligible LRTI presentations during the study period, of which 176 (26%) involved a CRP test. Three control practices recorded 258 presentations (based on the same eligibility criteria) from 35,928 patients.

The conversion of initial LRTI presentations to CRP tests (intervention arm only) and to other primary care healthcare events (both study arms) were reported descriptively. A binary outcome variable was created to represent antibiotic prescription during the 28 days following the initial LRTI presentation. As delayed prescribing was relatively infrequent at both intervention and control practices acute and delayed prescriptions were combined into a single outcome. Multivariate logistic regression was then used to estimate the odds of antibiotic prescription and follow-up consultation following initial presentation to practices in the intervention and control arms, adjusting for age (modelled as a binary variable with categories '< 44' and '≥ 45') and sex. Model fit was assessed using the likelihood ratio test, which indicated that patient sex was not a statistically significant predictor of either outcome. Adjusted odds ratios from the final models were reported, along with 95% confidence intervals and p-values to assess significance.

Overall, fewer initial presentations to intervention practices resulted in antibiotic prescription (59% of initial presentations, as compared to 79%) and follow-up consultations (30% compared to 38%), although there was little difference to antibiotic prescribing at follow-up (both arms 68%) (**table 2**). Furthermore, initial presentations with antibiotic prescription then resulting in follow-up consultation with an additional prescription were more common amongst control practices (21% compared to 13%).

	Intervention arm (n = 682)		Control arm (n = 258)	
	Outcome events	%	Outcome events	%
**CRP test at initial presentation**	176	26	-	-
**Antibiotic prescription at initial presentation**	405	59	204	79
**Follow-up consultation after initial presentation**	206	30	99	38
**Antibiotic prescription at follow-up consultation**	140	68 [a]	67	68 [a]
**Initial presentation with antibiotic prescription, then follow-up consultation with additional antibiotic prescription**	92	13	55	21

Table 2: Primary care healthcare events resulting from initial LRTI presentation
All percentages compared to number of initial presentations, except ([a]) compared to number of follow up consultations

We found that the odds of antibiotic prescribing after initial presentation were reduced by 62% amongst intervention practices, and the odds of follow up consultation were reduced by 32% (**table 3**). In each case we found that the outcome was more likely amongst presenting patients in the older age category.

Outcome	Variable	Adjusted OR (95% CI)	p-value
**Antibiotic prescription after initial presentation**	**Study arm**		
	Control	*Reference*	< 0.001
	Intervention	0.38 (0.27, 0.53)	
	**Patient age**		
	< 44	*Reference*	0.035
	≥ 45	1.35 (1.02, 1.77)	
**Follow-up consultation after initial presentation**	**Study arm**		
	Control	*Reference*	0.013
	Intervention	0.68 (0.51, 0.92)	
	**Patient age**		
	< 44	*Reference*	0.019
	≥ 45	1.40 (1.06, 1.85)	

Table 3: Multivariate logistic regression models for the association of practice-level intervention with antibiotic prescribing after initial presentation and follow-up consultation after initial presentation
