# Supplementary material for: Funding and policy incentives to encourage implementation of point-of-care C-reactive protein testing for lower respiratory tract infection in NHS primary care: a mixed-methods evaluation
Source: BMJ Open. 2018 Oct 25;8(10):e024558. doi: 10.1136/bmjopen-2018-024558 (PMC6224752; doi:10.1136/bmjopen-2018-024558)
Supplement: Author's manuscript [file bmjopen-2018-024558.draft_revisions.pdf]

BMJ Open is committed to open peer review. As part of this commitment we make the peer review history of every article we publish publicly available.

When an article is published we post the peer reviewers' comments and the authors' responses online. We also post the versions of the paper that were used during peer review. These are the versions that the peer review comments apply to.

The versions of the paper that follow are the versions that were submitted during the peer review process. They are not the versions of record or the final published versions. They should not be cited or distributed as the published version of this manuscript.

BMJ Open is an open access journal and the full, final, typeset and author-corrected version of record of the manuscript is available on our site with no access controls, subscription charges or pay-per-view fees (http://bmjopen.bmj.com).

If you have any questions on BMJ Open's open peer review process please email info.bmjopen@bmj.com

BMJ Open

Funding and policy incentives to encourage implementation of point-of-care C-reactive protein testing for lower respiratory tract infection in NHS primary care: a mixed-methods evaluation

Journal:	*BMJ Open*
Manuscript ID	bmjopen-2018-024558
Article Type:	Research
Date Submitted by the Author:	01-Jun-2018

Complete List of Authors:	Johnson, Matthew; Faculty of Health Sciences, University of Southampton, NIHR CLAHRC Wessex Data Science Hub<br>Cross, Liz; NIHR CLARHC East of England, Attenborough Surgery, Bushey Medical Centre, Herts Valleys Clinical Commissioning Group<br>Sandison, Nick; Faculty of Health Sciences, University of Southampton, NIHR CLAHRC Wessex<br>Stevenson, Jamie; Faculty of Health Sciences, University of Southampton, NIHR CLAHRC Wessex<br>Monks, T; Faculty of Health Sciences, University of Southampton, NIHR CLAHRC Wessex Data Science Hub<br>Moore, Michael; University of Southampton Medical School, Primary Care and Population Sciences

Keywords:	Health policy < HEALTH SERVICES ADMINISTRATION & MANAGEMENT, PRIMARY CARE, Respiratory infections < THORACIC MEDICINE

For peer review only

SCHOLARONE™
Manuscripts

Funding and policy incentives to encourage implementation of point-of-care C-reactive protein testing for lower respiratory tract infection in NHS primary care: a mixed-methods evaluation

Matthew Johnson

Matt.Johnson@soton.ac.uk

NIHR CLAHRC Wessex Data Science Hub, Faculty of Health Sciences, University of Southampton, UK

Liz Cross

liz.cross@nhs.net

NIHR CLARHC East of England, Attenborough Surgery, Bushey Medical Centre, Herts Valleys Clinical Commissioning Group, UK

Nick Sandison

N.J.Sandison@soton.ac.uk

NIHR CLAHRC Wessex, Faculty of Health Sciences, University of Southampton, UK

Jamie Stevenson

jamie.stevenson@soton.ac.uk

NIHR CLAHRC Wessex, Faculty of Health Sciences, University of Southampton, UK

Thomas Monks

thomas.monks@soton.ac.uk

NIHR CLAHRC Wessex Data Science Hub, Faculty of Health Sciences, University of Southampton, UK

Michael Moore (corresponding author)

mvm198@soton.ac.uk

Head of Academic Unit, Primary Care and Population Sciences, University of Southampton

For peer review only - http://bmjopen.bmj.com/site/about/guidelines.xhtml

Abstract

Objectives: Utilisation of point-of-care C-reactive protein testing for lower respiratory tract infection has been limited in UK primary care, with costs and funding suggested as important barriers. We aimed to use existing National Health Service funding and policy mechanisms to alleviate these barriers, and engage with clinicians and healthcare commissioners to encourage implementation.

Design: A mixed-methods study design was adopted, including a qualitative survey to identify clinicians' and commissioners' perceived benefits, barriers and enablers post-implementation, and quantitative analysis of results from a real-world implementation study.

Interventions: We developed a funding specification to underpin local reimbursement of general practices for test delivery based on an item of service payment. We also created training and administrative materials to facilitate implementation by reducing organisational burden. The implementation study provided intervention sites with a testing device and supplies, training and practical assistance.

Results: Despite engagement with several groups, implementation and uptake of our funding specification were limited. Survey respondents confirmed costs and funding as important barriers in addition to physical constraints and operational barriers, and cited training and the value of a local champion as enablers. The implementation study demonstrated reduced rates of antibiotic prescription and follow-up consultation amongst intervention sites.

Conclusions: Although survey respondents highlighted the clinical benefits, funding remains a barrier to implementation in UK primary care, and appears not to be alleviated by the existing financial incentives available to commissioners. The potential to meet incentive targets using lower cost methods, a lack of policy consistency, or competing financial pressures and commissioning programmes may be important determinants of local priorities. An implementation champion could help to catalyse support and overcome operational barriers at the local level, but widespread implementation is likely

For peer review only - http://bmjopen.bmj.com/site/about/guidelines.xhtml

to require national policy change. Successful implementation may reproduce antibiotic prescribing reductions observed in research studies.

Strengths and limitations of this study

- Use of a mixed-methods study design to assess the benefits, barriers and enablers of implementation from multiple perspectives.
- The study did not involve research funding for participating sites to enable evaluation of the impact of real-world financial structures associated with NHS commissioning.
- Development of a pack of resources that could contribute to future implementation projects.
- The study was undertaken against a background of general financial constraint within the NHS, which may have adversely impacted upon outcomes.

Background

Acute uncomplicated lower respiratory tract infection (LRTI) is the one of the commonest acute illnesses managed in primary care, and even in low antibiotic prescribing countries most patients receive antibiotics[1 2]. There is a clear national and international agenda to reduce unnecessary antibiotic prescribing[3]. The recently updated Cochrane review[4] of antibiotics for acute bronchitis demonstrated modest benefits, with a reduction of cough duration of around half a day. These findings were not replicated in a recently published large trial of antibiotics against placebo[5]. Limited benefit was demonstrated from antibiotics likely to be balanced by harms, and no subgroup was identified in whom there was a clinically relevant benefit[5 6].

In the absence of clear benefit then what are the drivers of continued prescribing? Patients are concerned about their symptoms[7], and clinicians are worried about missing severe infection and to avoid medico-legal consequences[7-9]. However, continued prescribing of antibiotics carries direct prescribing costs, increased re-consultations[10] and the major threat of antibiotic resistance[11]. Moreover,

For peer review only - http://bmjopen.bmj.com/site/about/guidelines.xhtml

a large cohort study has shown that adverse events following primary care consultation with LRTI patients are rare, and may not be directly influenced by prescribing strategy[12].

There is evidence that antibiotic prescribing in LRTI may be limited by appropriate use of near patient tests (NPT)[13-15]. Two candidates are available: C-reactive protein (CRP) and procalcitonin (PCT)[16-18]. An individual patient data review and meta-analysis supported the use of PCT to guide antibiotic use in acute settings including primary care, emergency units and intensive care, and demonstrated equivalent clinical outcomes with reduced antibiotic uptake[19]. Similarly, a recent Cochrane review examining the role of CRP in acute respiratory illness in primary care[20] included six trials with 3,284 participants and demonstrated a reduction in antibiotic use, although the results were interpreted with caution due to a high degree of heterogeneity. The recently published National Institute for Clinical Excellence (NICE) pneumonia guidelines[21] have also endorsed the use of CRP to aid decision making in primary care, selecting this ahead of PCT given the current non-availability of an NPT for PCT.

Several trials have explored the use of CRP in the primary care setting for management of LRTI, either alone or in combination with a communications skills training package, and have demonstrated a substantial reduction in antibiotic prescribing[13-15]. Although CRP is widely used in Scandinavian countries uptake has been limited in the UK, despite evidence of effectiveness in trial contexts to direct rational prescribing for LRTI. There is some question, however, of the effectiveness of CRP once adopted in clinical practice; results of tests performed on those with upper respiratory tract infection were found to have been misinterpreted, and modest effects on prescribing described[22]. Some have questioned whether reduced antibiotic prescribing will be seen following implementation in low prescribing settings[23], while others have reported CRP being the main determinant of antibiotic prescription in observational cohorts[24].

The reasons for the delayed uptake of NPT in the UK are not clear. Tests to reduce diagnostic uncertainty were supported by primary care physicians in a multi-country study including the UK[25]. Although studies suggest that CRP is a cost-effective means of addressing LRTI in primary care, there is

For peer review only

For peer review only - http://bmjopen.bmj.com/site/about/guidelines.xhtml

evidence that concerns around costs and funding remain a barrier to widespread implementation[26]. As the UK National Health Service (NHS) model of primary care does not include item of service payments, implementation of Point of Care testing (PoC) outside of a research setting would generate additional work and costs for initial purchase, maintenance and consumables, whereas antibiotic prescriptions have no direct cost at practice level. One plausible way to increase utilisation of CRP PoC would be the introduction of an item of service payment for use of the test in management of LRTI. The NHS England General Medical Services contract, in addition to defining the scope of standard primary care services to be delivered by general practices, also includes provision for opt-in to the delivery of additional, ‘locally enhanced’ services (LES)[27]. This study was based on the hypothesis that the LES scheme may provide a mechanism to introduce a financial incentive to uptake of CRP PoC for the management of LRTI in an NHS primary care setting.

We aimed to evaluate the efficacy of an item of service payment framework introduced at the local level by way of the LES scheme as a means of encouraging implementation amongst clinicians and healthcare commissioners. We also aimed to work with other groups and localities to explore alternative approaches to implementation, and to identify the perceived benefits, barriers and enablers using a post-implementation survey.

Methods

Leveraging funding and policy incentives

Our work has concentrated on making use of the opportunities afforded by existing NHS funding and policy mechanisms to encourage implementation of CRP PoC in primary care. We did not provide any research funding to participating organisations to ensure that successful implementation was not artificial, and could potentially be reproduced by others in the context of the real-world financial structures and constraints associated with healthcare commissioning in the NHS. All work in this area was undertaken during 2015 and 2016.

For peer review only

For peer review only - http://bmjopen.bmj.com/site/about/guidelines.xhtml

We developed a standard LES specification to underpin local implementation, establishing a funding framework of reimbursement of general practices by Clinical Commissioning Groups (CCGs) for CRP PoC on a unit basis. In view of the importance given to budgetary concerns by commissioners considering CRP implementation[26], CCGs may be motivated by its potential to open access to national funding associated with achieving the NHS England 'Quality Premium' (QP) target for reduced antibiotic prescribing in primary care[28].

Our research group, NIHR CLAHRC Wessex, is funded by both the National Institute for Health Research (NIHR) and partner organisations (including CCGs) within the local health system. Partner funding contributions may be monetary, or comprised of research study involvement. Our locality covers nine CCGs, each of whom had the opportunity to fulfil this funding obligation by participating in a CRP implementation study, or similar research. As well as this benefit, there was further opportunity for any participation costs to be partially or fully offset if the QP was achieved as a result.

Engaging with the NHS

Using materials from the GRACE Intro study[15] we developed resources including an online training course for general practitioners on the use of CRP, a clinical audit form and patient information leaflet[29]. All resources were made available to interested organisations as a means of facilitating implementation by reducing the associated administrative burden.

We visited clinicians and healthcare commissioners in our locality to generate interest, and made presentations at locality events to promote the LES framework. We also attended an NIHR CLARHC Wessex showcase event to which local CCGs were invited. We followed up additional enquiries from other groups outside of our locality who were interested in CRP implementation by offering visits and presentations, and sharing the resources developed for our local study. Resources were shared with ten groups across the country.

For peer review only - http://bmjopen.bmj.com/site/about/guidelines.xhtml

156 **Post-implementation survey**

157 In August 2017, following our period of NHS engagement, we issued an electronic survey to a convenience sample of clinicians and commissioners who had expressed an interest in, or were known to have contributed to, CRP implementation projects. Overall, nineteen individuals were invited to participate, including healthcare commissioners, pharmacists, primary and secondary care clinicians, and public health professionals.

162 We adopted a qualitative approach to explore in more depth the factors motivating respondents' initial interest, their experience of the implementation process and perceived barriers and enablers. Survey questions were written in line with these underlying objectives as deductively generated main themes[30] (**box 1**).

We asked participants:

- What were your/your organisation's reasons for implementing CRP testing?
- What was your experience of implementing and using CRP testing, and what is happening now?
- Which aspects of the implementation worked well?
- What were the barriers to implementation and/or continued use?
- How did you overcome these barriers?
- What would have helped, or would help in the future to encourage continued use?
- What would facilitate the implementation process?
- What would be your recommendations for those looking to implement CRP testing in the future?

Box 1: Post-implementation survey questions

167 Following the method of thematic analysis described by Nowell and colleagues[31], three members of the research team (MJ, NS, TM) individually reviewed all survey responses to inductively identify more specific subthemes. Reviewers took a systematic and iterative approach to analysis, later using researcher triangulation to reach consensus.

For peer review only - http://bmjopen.bmj.com/site/about/guidelines.xhtml
For peer review only

Funded by an NHS England Innovation Challenge Prize, an implementation study was undertaken in Herts Valleys CCG to evaluate CRP utilisation over three winter months (November 2016 – January 2017) and in five general practices, purposively sampled using standardised practice-level prescribing data to target high and medium antibiotic prescribers. The study aimed to evaluate whether, compared to standard care, the availability of CRP PoC for LRTI in primary care was associated with reduced acute and follow-up antibiotic prescribing, and unscheduled primary care reattendances and healthcare contacts in the 28 days following presentation.

Participating practices received an intervention consisting of one testing device and supplies to perform 100 tests, training on the NICE guidelines and equipment use, a review visit and practical assistance from the study team where appropriate; all other costs were borne by the practice. Each practice was free to select an appropriate device location and means of operationalising patient flow based on the physical layout of the practice, available resources and staff skill mix.

In line with the NICE guidelines, patients aged 18-65 presenting to intervention practices with suspected LRTI of less than three weeks' duration where there was diagnostic uncertainty were eligible to receive a test. Eligibility was assessed by the clinician during patient consultation. Patients with acute pneumonia, pregnant, immunocompromised, terminally ill or under follow up for Chronic Obstructive Pulmonary Disease were excluded[21]. As the offer was made on clinician discretion, and the patient entitled to refuse, some eligible patients did not receive a test. However, all eligible patients presenting to intervention practices were included in the evaluation, irrespective of whether they received a test.

The five intervention practices were compared to three Herts Valleys CCG control practices of similar size and prescribing level, all of which continued to provide standard care. Control practices did not receive training. One member of the study team (LC) conducted a retrospective electronic search at control practices to identify new clinical consultations (during the same study period) with patients who met the CRP eligibility criteria. Presentations were identified using a set of Read codes[32] commonly used to record clinical activity related to LRTI in NHS primary care, and relevant information collected for analytical purposes.

Results from the implementation study are given in **Appendix A**.

Box 2: Herts Valleys CCG implementation study

Results

Adoption of the LES framework and implementation of CRP

Whilst there was initial interest in CRP PoC facilitated by use of the LES framework, ultimately no CCGs within the NIHR CLAHRC Wessex locality participated in implementation projects. CRP was under consideration by one local CCG as part of a range of measures that might contribute to achieving a 'Quality, Innovation, Productivity and Prevention' programme target around improving detection of pneumonia in primary care, with the aim of enabling earlier intervention and reducing

For peer review only - http://bmjopen.bmj.com/site/about/guidelines.xhtml

hospital admissions. The CCG had planned to implement CRP across all of its general practices, but concluded that the associated upfront capital cost was too substantial and did not proceed.

Another CCG outside of our locality was interested in more widespread CRP implementation based on antibiotic prescribing reductions observed during a pilot undertaken in a single general practice. Although ten testing devices were procured and were initially regularly used, declining utilisation in the face of operational barriers prompted the CCG to cease procurement of PoC consumables. Financial incentivisation by way of the LES framework was considered as a means of encouraging utilisation, but ultimately failed to reengage interest.

We are not aware of any other CCGs having adopted the LES framework, or having engaged in implementation projects.

Post-implementation survey

Of the nineteen individuals invited to participate, seven (37%) submitted full responses. Several subthemes emerged from inductive analysis, with a high level of consistency amongst respondents (**table 1**).

All respondents reported being organisationally motivated by the potential for CRP PoC to help reduce antibiotic prescribing, while some further specified a desire to reduce variation in prescribing rates amongst practices in their locality. However, respondents also described mixed clinician utilisation: while some regularly incorporated PoC into consultations for suspected LRTI, others did not use it at all. Furthermore, one respondent noted that while utilisation had initially been high, it had declined over time.

For peer review only

Benefits:
• A clinical aid to appropriate antibiotic prescribing<br>• An objective measure to improve patient confidence in the prescribing action
Barriers:

For peer review only - http://bmjopen.bmj.com/site/about/guidelines.xhtml

• Limited time available during consultation<br>• Layout of facility and placement of testing device<br>• Cost of implementation and continued use<br>• Source of funding<br>• Resistance to change<br>• Maintaining engagement
Enablers:
• Early adopters to share experience and provide mentorship<br>• Training and education<br>• Champions within practice/locality<br>• Collaboration at local and national level<br>• Better utilisation of IT to facilitate testing process
**Table 1: Benefits, barriers and enablers of implementation**

Benefits

Most respondents agreed that CRP is a valuable clinical aid to appropriate antibiotic prescribing for patients with symptoms of LRTI. Furthermore, some highlighted its value as an objective measure to improve patient confidence in the chosen prescribing action, particularly in consultation with those who are “very keen” to receive antibiotics. Two respondents noted that, in their experience, patients had responded positively to the test and were satisfied with the outcome.

Barriers

In general, respondents reported that interest amongst clinicians was sometimes poor, and suggested a need for financial incentives and support to encourage widespread uptake. Most mentioned cost pressures, while some questioned who should be responsible for funding: general practices or the CCG. Despite the evidence base for the clinical benefits, one respondent suggested that there remains a need to “clearly demonstrate short term benefits in costs, workload and safety” to develop and maintain engagement.

Most respondents commented on the impact of operational constraints, such as the physical layout of the practice, how to accommodate multiple users, and the time required to carry out the test, particularly in the context of high workload and limited consultation duration. Although some respondents argued that other benefits justified its use despite these barriers, others specifically

For peer review only

cited them as disincentives, especially for clinicians who may have a negative attitude to CRP or be resistant to change.

Enablers

Most respondents discussed the importance of collaboration, although interpretations of this differed. Some suggested that early adopter sites share lessons learned to help others and avoid duplicated effort. The value of training and education during the implementation process were consistently emphasised, and development of a standard programme was suggested. Others mentioned the role of NIHR in fostering collaborative working, and the potential for general practice or CCG champions to improve engagement and resolve problems. Some respondents also suggested better use of IT to facilitate testing. Specific examples included the deployment of standard templates to record the test and result in the practice management system, and use of electronic alerts during consultation to prompt clinicians to PoC if indicated.

Discussion

Summary of main findings

Despite initial interest, there was no implementation in the NIHR CLAHRC Wessex locality, and no CCGs formally adopted the LES framework. The research team were unable to gain significant traction with CCG management, and when contact was established CCGs were unwilling to prioritise antibiotic stewardship over other local initiatives. The policy levers seemed to have little impact in this locality, where CCGs were struggling to remain in budget. The financial rewards arising from the QP only applied to CCGs meeting financial targets. Elsewhere, one CCG implemented CRP and, following declining utilisation in response to operational barriers, found that the LES framework was insufficient as a mechanism to reengage interest.

Although the small sample size limits inference and generalisability, our post-implementation survey identified several financial, operational and physical barriers in common with previous qualitative

For peer review only - http://bmjopen.bmj.com/site/about/guidelines.xhtml

research[26]. Respondents confirmed that implementation would be unlikely without financial incentives but also highlighted difficulties integrating PoC into practice workflow, and constraints arising from a lack of dedicated space, equipment sharing and limited time. Reported enablers included adequate training and the value of a local champion.

Some respondents also emphasised the clinical benefits of CRP, giving anecdotal examples of cases where testing had prevented antibiotic prescription. The potential for more widespread repetition of this outcome is suggested by quantitative results from the Herts Valley CCG implementation study, where a successful, separately funded implementation scheme was run for a three month period, driven by a local champion. Observation of substantial prescribing reductions amongst intervention practices suggests that implementation in the NHS might replicate the prescribing reductions reported in research studies.

Comparison with other literature

We are unaware of any other implementation studies concerning CRP PoC in the UK. In other health settings PoC is widely adopted[22], and following government directives has been introduced in the Netherlands[33]. The financial barriers to implementation have been identified in a previous study including European and UK participants[23], which noted that countries with high rates of use had alternative reimbursement models, and that widespread implementation in Europe followed health policy change. The same study also highlighted issues around workflow and time as potential barriers to implementation in the UK.

Strengths and weaknesses

Our study describes the results of attempts at CRP implementation without the resources associated with research, and without specific policy directives. It is unclear how generalisable our findings might be; it would appear that CCG partnership with NIHR CLAHRC Wessex and national level incentives via the QP should have maximised the potential for local implementation. The scheme

For peer review only

For peer review only - http://bmjopen.bmj.com/site/about/guidelines.xhtml

was devised during a time of general financial constraint within the NHS, which may have had particular impact in the Wessex locality.

Limitations around funding mechanisms

The criteria required to achieve the QP, even taking the antibiotic prescribing element alone, has been inconsistent. Some changes have been significant, such as a move to greater emphasis on antibiotic prescribing for urinary tract infection as of 2018/19[28]. Furthermore, as the QP is awarded retrospectively and is contingent upon meeting other financial targets, the funding mechanism is not guaranteed, making it difficult to engage commissioners and to create a firm financial framework to underpin CRP implementation.

A further feature of the QP is that no method of achievement is stipulated; the antibiotic prescribing element simply requires an absolute prescribing reduction. The NHS has reported a national ~7% reduction in primary care coinciding with the implementation phase of this study[34-36], which may have resulted from a general policy shift and increased focus of clinical training in primary care. This suggests that overall improvements could be gained and the QP target potentially achieved by way of alternative, lower cost methods alone, negating commissioners' financial incentive for CRP implementation irrespective of the clinical benefits.

The pressures of multiple, competing commissioning programmes may limit engagement with certain initiatives, while the overall funding structure of the NHS may also influence commissioners' preferences and priorities. One CCG within our locality suggested that, despite evidence of a net cost saving associated with CRP[37], whilst the upfront implementation costs reside with primary care, any savings would principally be realised by the secondary care sector. In this instance, therefore, concerns that the costs and benefits of the initiative may be distinctly localised within separate areas of the health system acted as a disincentive to its adoption.

Implications

For peer review only - http://bmjopen.bmj.com/site/about/guidelines.xhtml

Whilst the use of existing financial structures appeared appealing as a mechanism, it was not possible to fully test the hypothesis that modest financial incentives to general practices at local level would enable CRP implementation, as financial pressures impeded CCG adoption of the policy. National incentives for CCGs did not appear to override the financial constraints because a) financial rewards were only available to CCGs meeting financial targets, and b) antibiotic targets were being achieved through other mechanisms not requiring financial investment.

Although a small case study suggests that implementation outside of research studies may result in similar prescribing reductions, since it was driven by local investment and a local champion it may not fully reflect implementation in routine practice, or be generalisable to other areas. Furthermore, and recalling questions over the primacy of lower cost measures, the fact that this intervention provided training and support in addition to testing materials limits the extent to which the observed prescribing reductions can be confidently attributed to CRP PoC alone.

The value of an enthusiastic, local champion to catalyse support for implementation emerged from both the qualitative and quantitative strands of this study. Knowledge mobilisation and implementation in practice may be assisted by way of a Researcher-In-Residence model[38], while further qualitative and observational research could improve understanding of how champions are able to persuade and engage clinicians and to encourage commissioners to look beyond the immediate financial disincentives, and whether they may be effective in other areas and settings. Further economic research might also model different modes of implementation to assess the costs and consequences across the system, and to find alternative funding models to overcome the financial barriers. Multi-purpose testing devices, for example, may have the advantage of spreading investment across several funding streams.

In conclusion, it seems unlikely that financial schemes falling outside of national policy will gain much traction in a financially constrained NHS. Full-scale implementation of CRP PoC is likely to require central implementation via government policy or contractual changes.

For peer review only

Appendix A: Results from Herts Valleys CCG implementation study

Five intervention practices with a total list size of 63,743 patients recorded 682 eligible LRTI presentations during the study period, of which 176 (26%) involved a CRP test. Three control practices recorded 258 presentations (based on the same eligibility criteria) from 35,928 patients.

Overall, fewer initial presentations to intervention practices resulted in antibiotic prescription over the following 28 days (59% of initial presentations, as compared to 79%) and follow-up consultations (30% compared to 38%), although there was little difference to antibiotic prescribing at follow-up (both arms 68%) (**table 2**). Furthermore, initial presentations with antibiotic prescription then resulting in follow-up consultation with an additional prescription were more common amongst control practices (21% compared to 13%). As delayed prescribing was relatively infrequent at both intervention and control practices all prescriptions were combined into a single outcome, irrespective of delay.

	**Intervention arm (n = 682)**		**Control arm (n = 258)**	
	Outcome events	%	Outcome events	%
**CRP test at initial presentation**	176	26	-	-
**Antibiotic prescription at initial presentation**	405	59	204	79
**Follow-up consultation after initial presentation**	206	30	99	38
**Antibiotic prescription at follow-up consultation**	140	68 [a]	67	68 [a]
**Initial presentation with antibiotic prescription, then follow-up consultation with additional antibiotic prescription**	92	13	55	21

Table 2: Primary care healthcare events resulting from initial LRTI presentation
All percentages compared to number of initial presentations, except ([a]) compared to number of follow up consultations

Using logistic regression, we found that the odds of antibiotic prescribing at initial presentation (where acute and delayed prescribing were grouped into a single outcome) were reduced by 62% amongst intervention practices, and the odds of follow up consultation reduced by 32% (**table 3**). In each case we adjusted for patient age (modelled as a binary variable with categories ‘< 44’ and ‘≥ 45’), and found that each outcome was more likely amongst presenting patients in the older age category.

For peer review only

Outcome	Variable	Adjusted OR (95% CI)	p-value
**Antibiotic prescription at initial presentation**	**Study arm**		< 0.001
	Control	*Reference*	
	Intervention	0.38 (0.27, 0.53)	
	**Patient age**		0.035
	< 44	*Reference*	
	≥ 45	1.35 (1.02, 1.77)	
**Follow-up consultation after initial presentation**	**Study arm**		0.013
	Control	*Reference*	
	Intervention	0.68 (0.51, 0.92)	
	**Patient age**		0.019
	< 44	*Reference*	
	≥ 45	1.40 (1.06, 1.85)	

Table 3: Multivariate logistic regression models for the association of practice-level intervention with antibiotic prescribing at initial presentation and follow-up consultation after initial presentation

For peer review only

List of abbreviations

LRTI: Lower respiratory tract infection; NPT: Near patient test; CRP: C-reactive protein; PCT: Procalcitonin; NICE: National Institute for Clinical Excellence; NHS: National Health Service; PoC: Point of care testing; LES: Locally Enhanced Service; CCG: Clinical Commissioning Group; QP: NHS England Quality Premium; NIHR: National Institute for Health Research

Declarations

Ethical approval

The Integrated Research Application System (IRAS) confirmed that formal ethical approval was not required for the Herts Valleys CCG implementation study; a service evaluation project.

Funding statement

This research was funded by the National Institute for Health Research (NIHR) Collaboration for Leadership in Applied Health Research and Care Wessex at University Hospital Southampton NHS Foundation Trust. The views expressed are those of the author(s) and not necessarily those of the NHS, the NIHR or the Department of Health and Social Care.

For peer review only - http://bmjopen.bmj.com/site/about/guidelines.xhtml

The Herts Valleys CCG implementation study was separately supported by a 2015/16 NHS England Innovation Challenge Prize, Acorn award.

Competing interests

MJ, NS, JS, TM and MM have no competing interests to declare. LC has received honoraria from Abbott Laboratories and Roche Diagnostics Ltd for speaking events.

Authors' contributions

MJ carried out quantitative analysis and wrote the paper with contributions from MM. MJ and NS developed the post-implementation survey and, with TM, carried out qualitative analysis. LC carried out the Herts Valleys CCG implementation study. JS, NS and MM developed the LES framework and other resources, and engaged with the NHS. TM provided methodological input and MM provided clinical guidance. All authors commented on drafts of the paper and have read and approved the final manuscript.

Patient consent for publication

Not applicable.

Data sharing statement

Not applicable.

Acknowledgements

The authors thank Elsie Griffins and Dr Denise Knight for their support for the Herts Valleys CCG implementation study.

References

1. Butler CC, Hood K, Verheij T, et al. Variation in antibiotic prescribing and its impact on recovery in patients with acute cough in primary care: prospective study in 13 countries. *BMJ* 2009;338(b2242)
2. Akkerman AE, van der Wouden JC, Kuyvenhoven MM, et al. Antibiotic prescribing for respiratory tract infections in Dutch primary care in relation to patient age and clinical entities. *J Antimicrob Chemother* 2004;54(6):1116-21. doi: 10.1093/jac/dkh480 [published Online First: 2004/11/18]

For peer review only - http://bmjopen.bmj.com/site/about/guidelines.xhtml

For peer review only

3. Davies SC. Annual Report of the Chief Medical Officer, Volume Two, 2011, Infections and the rise of antimicrobial resistance. London: Department of Health 2013.
4. Smith SM, Fahey T, Smucny J, et al. Antibiotics for acute bronchitis. *Cochrane Database Syst Rev* 2014(3):CD000245. doi: 10.1002/14651858.CD000245.pub3 [published Online First: 2014/03/04]
5. Little P, Stuart B, Moore M, et al. Amoxicillin for acute lower-respiratory-tract infection in primary care when pneumonia is not suspected: a 12-country, randomised, placebo-controlled trial. *The Lancet Infectious Diseases* 2013;13(2):123-29. doi: 10.1016/s1473-3099(12)70300-6
6. Moore M, Stuart B, Coenen S, et al. Amoxicillin for acute lower respiratory tract infection in primary care: subgroup analysis of potential high-risk groups. *Br J Gen Pract* 2014;64(619):e75-80. doi: 10.3399/bjgp14X677121 [published Online First: 2014/02/26]
7. Cornford CS. Why patients consult when they cough: a comparison of consulting and non-consulting patients. *British Journal of General Practice* 1998;48(436):1751-4.
8. Little P, Watson L, Morgan S, et al. Antibiotic prescribing and admissions with major suppurative complications of respiratory tract infections: a data linkage study. *British Journal of General Practice* 2002;52:187-93.
9. Winchester CC, Macfarlane TV, Thomas M, et al. Antibiotic prescribing and outcomes of lower respiratory tract infection in UK primary care. *Chest* 2009;135(5):1163-72. doi: 10.1378/chest.07-2940 [published Online First: 2009/05/08]
10. Moore M, Little P, Rumsby K, et al. Effect of antibiotic prescribing strategies and an information leaflet on longer-term reconsultation for acute lower respiratory tract infection. *Br J Gen Pract* 2009;59(567):728-34. doi: 10.3399/bjgp09X472601 [published Online First: 2009/10/22]
11. Goossens H, Ferech M, Vander Stichele R, et al. Outpatient antibiotic use in Europe and association with resistance: a cross-national database study. *The Lancet* 2005;365(9459):579-87. doi: 10.1016/s0140-6736(05)70799-6
12. Little P, Stuart B, Smith S, et al. Antibiotic prescription strategies and adverse outcome for uncomplicated lower respiratory tract infections: prospective cough complication cohort (3C) study. *BMJ* 2017;357:j2148. doi: 10.1136/bmj.j2148 [published Online First: 2017/05/24]
13. Cals JW, Butler CC, Hopstaken RM, et al. Effect of point of care testing for C reactive protein and training in communication skills on antibiotic use in lower respiratory tract infections: cluster randomised trial. *BMJ* 2009;338:b1374. doi: 10.1136/bmj.b1374 [published Online First: 2009/05/07]
14. Cals JW, Schot MJ, de Jong SA, et al. Point-of-care C-reactive protein testing and antibiotic prescribing for respiratory tract infections: a randomized controlled trial. *Ann Fam Med* 2010;8(2):124-33. doi: 10.1370/afm.1090 [published Online First: 2010/03/10]
15. Little P, Stuart B, Francis N, et al. Effects of internet-based training on antibiotic prescribing rates for acute respiratory-tract infections: a multinational, cluster, randomised, factorial, controlled trial. *The Lancet* 2013;382(9899):1175-82. doi: 10.1016/s0140-6736(13)60994-0
16. Briel M, Schuetz P, Mueller B, et al. Procalcitonin-Guided Antibiotic Use vs a Standard Approach for Acute Respiratory Tract Infections in Primary Care. *Arch Intern Med* 2008;168(18):2000-07.
17. Holm A, Pedersen SS, Nexoe J, et al. Procalcitonin versus C-reactive protein for predicting pneumonia in adults with lower respiratory tract infection in primary care. *British Journal of General Practice* 2007;57:555-60.
18. Christ-Crain M, Opal SM. Clinical review: The role of biomarkers in the diagnosis and management of community acquired pneumonia. *Critical Care* 2010;14(203) doi: 10.1186/cc8155
19. Schuetz P, Briel M, Christ-Crain M, et al. Procalcitonin to guide initiation and duration of antibiotic treatment in acute respiratory infections: an individual patient data meta-analysis.

For peer review only - http://bmjopen.bmj.com/site/about/guidelines.xhtml

Clin Infect Dis 2012;55(5):651-62. doi: 10.1093/cid/cis464 [published Online First: 2012/05/11]
20. Aabenhus R, Jensen JU, Jorgensen KJ, et al. Biomarkers as point-of-care tests to guide prescription of antibiotics in patients with acute respiratory infections in primary care. *Cochrane Database Syst Rev* 2014(11):CD010130. doi: 10.1002/14651858.CD010130.pub2 [published Online First: 2014/11/07]
21. National Institute for Health and Care Excellence. Pneumonia in adults: diagnosis and management (CG191) 2014 [Available from: https://www.nice.org.uk/guidance/cg191 accessed 13 March 2018.
22. André M, Schwan A, Odenholt I. The Use of CRP Tests in Patients with Respiratory Tract Infections in Primary Care in Sweden Can Be Questioned. *Scandinavian Journal of Infectious Diseases* 2009;36(3):192-97. doi: 10.1080/00365540410019372
23. Minnaard MC, van de Pol AC, Hopstaken RM, et al. C-reactive protein point-of-care testing and associated antibiotic prescribing. *Fam Pract* 2016;33(4):408-13. doi: 10.1093/fampra/cmw039 [published Online First: 2016/05/28]
24. Jakobsen KA, Melbye H, Kelly MJ, et al. Influence of CRP testing and clinical findings on antibiotic prescribing in adults presenting with acute cough in primary care. *Scand J Prim Health Care* 2010;28(4):229-36. doi: 10.3109/02813432.2010.506995 [published Online First: 2010/08/14]
25. Tonkin-Crine S, Yardley L, Coenen S, et al. GPs' views in five European countries of interventions to promote prudent antibiotic use. *Br J Gen Pract* 2011;61(586):e252-61. doi: 10.3399/bjgp11X572445 [published Online First: 2011/05/31]
26. Huddy JR, Ni MZ, Barlow J, et al. Point-of-care C reactive protein for the diagnosis of lower respiratory tract infection in NHS primary care: a qualitative study of barriers and facilitators to adoption. *BMJ Open* 2016;6(3):e009959. doi: 10.1136/bmjopen-2015-009959 [published Online First: 2016/03/05]
27. NHS England. NHS England Standard General Medical Services Contract 2017/18 2018 [Available from: https://www.england.nhs.uk/gp/gpfv/investment/gp-contract/ accessed 13 March 2018.
28. NHS England. Quality Premium 2018 [Available from: https://www.england.nhs.uk/resources/resources-for-ccgs/ccg-out-tool/ccg-ois/qual-prem/ accessed 16 May 2018.
29. NIHR CLAHRC Wessex. Reducing unnecessary antibiotic prescribing in primary care 2018 [Available from: https://www.clahrc-wessex.nihr.ac.uk/theme/project/20 accessed 24th May 2018.
30. Braun V, Clarke V. Using thematic analysis in psychology. *Qualitative Research in Psychology* 2006;3(2):77-101. doi: 10.1191/1478088706qp063oa
31. Nowell LS, Norris JM, White DE, et al. Thematic Analysis: Striving to Meet the Trustworthiness Criteria. *International Journal of Qualitative Methods* 2017;16:1-13. doi: 10.1177/1609406917733847
32. Benson T. The history of the Read codes: the inaugural James Read Memorial Lecture 2011. *Journal of Innovation in Health Informatics* 2011;19(3):173-82. doi: 10.14236/jhi.v19i3.811
33. The Dutch College of General Practitioners (NHG). NHG-Standaard Acuut hoesten (Acute cough guidelines) 2018 [Available from: https://www.nhg.org/standaarden/volledig/nhg-standaard-acuut-hoesten accessed 31st May 2018.
34. NHS England. Success in NHS push to reduce avoidable antibiotic prescribing 2016 [Available from: https://www.england.nhs.uk/2016/03/antibiotic-prescribing/ accessed 17 May 2018.
35. NHS England. Antibiotic quality premium monitoring dashboard 2018 [Available from: https://www.england.nhs.uk/resources/resources-for-ccgs/ccg-out-tool/ccg-ois/anti-dash/ accessed 17 May 2018.

For peer review only - http://bmjopen.bmj.com/site/about/guidelines.xhtml

36. NHS Improvement. Helping GPs to cut antibiotic prescriptions by 2.6m in just one year 2016 [Available from: https://improvement.nhs.uk/news-alerts/helping-gps-cut-antibiotic-prescriptions/ accessed 17 May 2018.
37. Hunter R. Cost-effectiveness of point-of-care C-reactive protein tests for respiratory tract infection in primary care in England. *Adv Ther* 2015;32(1):69-85. doi: 10.1007/s12325-015-0180-x [published Online First: 2015/01/27]
38. Marshall M, Pagel C, French C, et al. Moving improvement research closer to practice: the Researcher-in-Residence model. *BMJ Qual Saf* 2014;23(10):801-5. doi: 10.1136/bmjqs-2013-002779 [published Online First: 2014/06/05]

For peer review only

For peer review only - http://bmjopen.bmj.com/site/about/guidelines.xhtml

STROBE 2007 (v4) checklist of items to be included in reports of observational studies in epidemiology*
Checklist for cohort, case-control, and cross-sectional studies (combined)

Section/Topic	Item #	Recommendation	Reported on page #
**Title and abstract**	1	(*a*) Indicate the study's design with a commonly used term in the title or the abstract	Mixed-methods design reported in title (page 1), and described in more detail in abstract (page 2)
		(*b*) Provide in the abstract an informative and balanced summary of what was done and what was found	Page 2-3
**Introduction**			
Background/rationale	2	Explain the scientific background and rationale for the investigation being reported	Page 3-5
Objectives	3	State specific objectives, including any pre-specified hypotheses	Page 5
**Methods**			
Study design	4	Present key elements of study design early in the paper	Described briefly in abstract (page 2), and in more detail in methods (page 5-8)
Setting	5	Describe the setting, locations, and relevant dates, including periods of recruitment, exposure, follow-up, and data collection	Separately reported for engagement work (page 5-6), qualitative component (page 7), and quantitative component (page 8)
Participants	6	(*a*) *Cohort study*—Give the eligibility criteria, and the sources and methods of selection of participants. Describe methods of follow-up<br>*Case-control study*—Give the eligibility criteria, and the sources and methods of case ascertainment and control selection. Give the rationale for the choice of cases and controls<br>*Cross-sectional study*—Give the eligibility criteria, and the sources and methods of selection of participants	Separately reported for engagement work (page 5-6), qualitative component (page 7), and quantitative component (page 8)
		(*b*) *Cohort study*—For matched studies, give matching criteria and number of exposed and unexposed<br>*Case-control study*—For matched studies, give matching criteria and the number of controls per case	Not applicable. Quantitative component used intervention/control arms, but not matched; purposive sample and basis for comparison described (page 8)
Variables	7	Clearly define all outcomes, exposures, predictors, potential confounders, and effect modifiers. Give diagnostic criteria, if applicable	Described for quantitative component (page 15)
Data sources/ measurement	8*	For each variable of interest, give sources of data and details of methods of assessment (measurement).	Described for quantitative

For peer review only

		Describe comparability of assessment methods if there is more than one group	component (page 8)
Bias	9	Describe any efforts to address potential sources of bias	Acknowledged potential bias arising from quantitative component (page 14), however not possible to quantify and address in analysis
Study size	10	Explain how the study size was arrived at	Recruitment of participants/study size separately reported for engagement work (page 6), qualitative component (page 7), and quantitative component (page 8)
Quantitative variables	11	Explain how quantitative variables were handled in the analyses. If applicable, describe which groupings were chosen and why	Reported for quantitative component (page 15)
Statistical methods	12	(*a*) Describe all statistical methods, including those used to control for confounding	Reported for quantitative component (page 15)
		(*b*) Describe any methods used to examine subgroups and interactions	Not applicable
		(*c*) Explain how missing data were addressed	Not applicable
		(*d*) *Cohort study*—If applicable, explain how loss to follow-up was addressed<br>*Case-control study*—If applicable, explain how matching of cases and controls was addressed<br>*Cross-sectional study*—If applicable, describe analytical methods taking account of sampling strategy	Not applicable
		(*e*) Describe any sensitivity analyses	Not applicable
**Results**			
Participants	13*	(a) Report numbers of individuals at each stage of study—eg numbers potentially eligible, examined for eligibility, confirmed eligible, included in the study, completing follow-up, and analysed	Reported for quantitative component (page 15), including eligibility criteria (page 8)
		(b) Give reasons for non-participation at each stage	Not applicable
		(c) Consider use of a flow diagram	Not applicable
Descriptive data	14*	(a) Give characteristics of study participants (eg demographic, clinical, social) and information on exposures and potential confounders	Not applicable
		(b) Indicate number of participants with missing data for each variable of interest	Not applicable
		(c) *Cohort study*—Summarise follow-up time (eg, average and total amount)	Reported duration of quantitative component follow-up period (page 8 and 15)

For peer review only

Outcome data	15*	*Cohort study*—Report numbers of outcome events or summary measures over time	Reported for quantitative component (page 15)
		Case-control study—Report numbers in each exposure category, or summary measures of exposure	Not applicable
		Cross-sectional study—Report numbers of outcome events or summary measures	Not applicable
Main results	16	(*a*) Give unadjusted estimates and, if applicable, confounder-adjusted estimates and their precision (eg, 95% confidence interval). Make clear which confounders were adjusted for and why they were included	Quantitative analysis reported briefly in appendix (page 15). Adjusted odds ratios presented, with confidence intervals and confounding variables
		(*b*) Report category boundaries when continuous variables were categorized	Reported for quantitative component (page 15)
		(*c*) If relevant, consider translating estimates of relative risk into absolute risk for a meaningful time period	Not applicable
Other analyses	17	Report other analyses done—eg analyses of subgroups and interactions, and sensitivity analyses	Not applicable
**Discussion**			
Key results	18	Summarise key results with reference to study objectives	Reported for all study components throughout discussion (page 11-14)
Limitations	19	Discuss limitations of the study, taking into account sources of potential bias or imprecision. Discuss both direction and magnitude of any potential bias	Overall study limitations discussed (page 12), including specific acknowledgement of potential for bias arising from quantitative component (page 14)
Interpretation	20	Give a cautious overall interpretation of results considering objectives, limitations, multiplicity of analyses, results from similar studies, and other relevant evidence	Overall interpretation of results discussed (page 13-14)
Generalisability	21	Discuss the generalisability (external validity) of the study results	Statements on generalisability given (page 12, 14)
**Other information**			
Funding	22	Give the source of funding and the role of the funders for the present study and, if applicable, for the original study on which the present article is based	Page 16

*Give information separately for cases and controls in case-control studies and, if applicable, for exposed and unexposed groups in cohort and cross-sectional studies.

Note: An Explanation and Elaboration article discusses each checklist item and gives methodological background and published examples of transparent reporting. The STROBE checklist is best used in conjunction with this article (freely available on the Web sites of PLoS Medicine at http://www.plosmedicine.org/, Annals of Internal Medicine at http://www.annals.org/, and Epidemiology at http://www.epidem.com/). Information on the STROBE Initiative is available at www.strobe-statement.org.

For peer review only

BMJ Open

Funding and policy incentives to encourage implementation of point-of-care C-reactive protein testing for lower respiratory tract infection in NHS primary care: a mixed-methods evaluation

Journal:	*BMJ Open*
Manuscript ID	bmjopen-2018-024558.R1
Article Type:	Research
Date Submitted by the Author:	16-Jul-2018
Complete List of Authors:	Johnson, Matthew; Faculty of Health Sciences, University of Southampton, NIHR CLAHRC Wessex Data Science Hub<br>Cross, Liz; NIHR CLARHC East of England, Attenborough Surgery, Bushey Medical Centre, Herts Valleys Clinical Commissioning Group<br>Sandison, Nick; Faculty of Health Sciences, University of Southampton, NIHR CLAHRC Wessex<br>Stevenson, Jamie; Faculty of Health Sciences, University of Southampton, NIHR CLAHRC Wessex<br>Monks, T; Faculty of Health Sciences, University of Southampton, NIHR CLAHRC Wessex Data Science Hub<br>Moore, Michael; University of Southampton Medical School, Primary Care and Population Sciences
**Primary Subject Heading**:	General practice / Family practice
Secondary Subject Heading:	Health policy
Keywords:	Health policy < HEALTH SERVICES ADMINISTRATION & MANAGEMENT, PRIMARY CARE, Respiratory infections < THORACIC MEDICINE

SCHOLARONE™ Manuscripts

For peer review only - http://bmjopen.bmj.com/site/about/guidelines.xhtml

Funding and policy incentives to encourage implementation of point-of-care C-reactive protein testing for lower respiratory tract infection in NHS primary care: a mixed-methods evaluation

Matthew Johnson (corresponding author)

mj1c13@soton.ac.uk

NIHR CLAHRC Wessex Data Science Hub, Faculty of Health Sciences, University of Southampton, UK

Liz Cross

liz.cross@nhs.net

NIHR CLARHC East of England, Attenborough Surgery, Bushey Medical Centre, Herts Valleys Clinical Commissioning Group, UK

Nick Sandison

N.J.Sandison@soton.ac.uk

NIHR CLAHRC Wessex, Faculty of Health Sciences, University of Southampton, UK

Jamie Stevenson

jamie.stevenson@soton.ac.uk

NIHR CLAHRC Wessex, Faculty of Health Sciences, University of Southampton, UK

Thomas Monks

thomas.monks@soton.ac.uk

NIHR CLAHRC Wessex Data Science Hub, Faculty of Health Sciences, University of Southampton, UK

Michael Moore

mvm198@soton.ac.uk

Head of Academic Unit, Primary Care and Population Sciences, University of Southampton

For peer review only - http://bmjopen.bmj.com/site/about/guidelines.xhtml

Abstract

Objectives: Utilisation of point-of-care C-reactive protein testing for lower respiratory tract infection has been limited in UK primary care, with costs and funding suggested as important barriers. We aimed to use existing National Health Service funding and policy mechanisms to alleviate these barriers, and engage with clinicians and healthcare commissioners to encourage implementation.

Design: A mixed-methods study design was adopted, including a qualitative survey to identify clinicians' and commissioners' perceived benefits, barriers and enablers post-implementation, and quantitative analysis of results from a real-world implementation study.

Interventions: We developed a funding specification to underpin local reimbursement of general practices for test delivery based on an item of service payment. We also created training and administrative materials to facilitate implementation by reducing organisational burden. The implementation study provided intervention sites with a testing device and supplies, training and practical assistance.

Results: Despite engagement with several groups, implementation and uptake of our funding specification were limited. Survey respondents confirmed costs and funding as important barriers in addition to physical and operational constraints, and cited training and the value of a local champion as enablers.

Conclusions: Although survey respondents highlighted the clinical benefits, funding remains a barrier to implementation in UK primary care, and appears not to be alleviated by the existing financial incentives available to commissioners. The potential to meet incentive targets using lower cost methods, a lack of policy consistency, or competing financial pressures and commissioning programmes may be important determinants of local priorities. An implementation champion could help to catalyse support and overcome operational barriers at the local level, but widespread implementation is likely to require national policy change. Successful implementation may reproduce antibiotic prescribing reductions observed in research studies.

For peer review only - http://bmjopen.bmj.com/site/about/guidelines.xhtml

Strengths and limitations of this study

- Use of a mixed-methods study design to assess the benefits, barriers and enablers of implementation from multiple perspectives.
- The study did not involve research funding for participating sites to enable evaluation of the impact of real-world financial structures associated with NHS commissioning.
- Development of a pack of resources that could contribute to future implementation projects.
- The study was undertaken against a background of general financial constraint within the NHS, which may have adversely impacted upon outcomes.

Background

Acute uncomplicated lower respiratory tract infection (LRTI) is the one of the commonest acute illnesses managed in primary care, and even in low antibiotic prescribing countries most patients receive antibiotics[1 2]. There is a clear national and international agenda to reduce unnecessary antibiotic prescribing[3]. The recently updated Cochrane review[4] of antibiotics for acute bronchitis demonstrated modest benefits, with a reduction of cough duration of around half a day. These findings were not replicated in a recently published large trial of antibiotics against placebo[5]. Limited benefit was demonstrated from antibiotics likely to be balanced by harms, and no subgroup was identified in whom there was a clinically relevant benefit[5 6].

In the absence of clear benefit then what are the drivers of continued prescribing? Patients are concerned about their symptoms[7], and clinicians are worried about missing severe infection and to avoid medico-legal consequences[7-9]. However, continued prescribing of antibiotics carries direct prescribing costs, increased re-consultations[10] and the major threat of antibiotic resistance[11]. Moreover, a large cohort study has shown that adverse events following primary care consultation with LRTI patients are rare, and may not be directly influenced by prescribing strategy[12].

For peer review only - http://bmjopen.bmj.com/site/about/guidelines.xhtml

There is evidence that antibiotic prescribing in LRTI may be limited by appropriate use of near patient tests (NPT)[13-15]. Two candidates are available: C-reactive protein (CRP) and procalcitonin (PCT)[16-18]. An individual patient data review and meta-analysis supported the use of PCT to guide antibiotic use in acute settings including primary care, emergency units and intensive care, and demonstrated equivalent clinical outcomes with reduced antibiotic uptake[19]. Similarly, a recent Cochrane review examining the role of CRP in acute respiratory illness in primary care[20] included six trials with 3,284 participants and demonstrated a reduction in antibiotic use, although the results were interpreted with caution due to a high degree of heterogeneity. The recently published National Institute for Clinical Excellence (NICE) pneumonia guidelines[21] have also endorsed the use of CRP to aid decision making in primary care, selecting this ahead of PCT given the current non-availability of an NPT for PCT.

Several trials have explored the use of CRP in the primary care setting for management of LRTI, either alone or in combination with a communications skills training package, and have demonstrated a substantial reduction in antibiotic prescribing[13-15]. Although CRP is widely used in Scandinavian countries uptake has been limited in the UK, despite evidence of effectiveness in trial contexts to direct rational prescribing for LRTI. There is some question, however, of the effectiveness of CRP once adopted in clinical practice; results of tests performed on those with upper respiratory tract infection were found to have been misinterpreted, and modest effects on prescribing described[22]. Some have questioned whether reduced antibiotic prescribing will be seen following implementation in low prescribing settings[23], while others have reported CRP being the main determinant of antibiotic prescription in observational cohorts[24].

The reasons for the delayed uptake of NPT in the UK are not clear. Tests to reduce diagnostic uncertainty were supported by primary care physicians in a multi-country study including the UK[25]. Although studies suggest that CRP is a cost-effective means of addressing LRTI in primary care, there is evidence that concerns around costs and funding remain a barrier to widespread implementation[26]. As the UK National Health Service (NHS) model of primary care does not include item of service payments,

For peer review only

implementation of Point of Care testing (PoC) outside of a research setting would generate additional work and costs for initial purchase, maintenance and consumables, whereas antibiotic prescriptions have no direct cost at practice level (see **Appendix A** for further detail of primary care testing in the NHS system). One plausible way to increase utilisation of CRP PoC would be the introduction of an item of service payment for use of the test in management of LRTI. The NHS England General Medical Services contract, in addition to defining the scope of standard primary care services to be delivered by general practices, also includes provision for opt-in to the delivery of additional, 'locally enhanced' services (LES)[27]. This study was based on the hypothesis that the LES scheme may provide a mechanism to introduce a financial incentive to uptake of CRP PoC for the management of LRTI in an NHS primary care setting.

We aimed to evaluate the efficacy of an item of service payment framework introduced at the local level by way of the LES scheme as a means of encouraging implementation amongst clinicians and healthcare commissioners. We also aimed to work with other groups and localities to explore alternative approaches to implementation, and to identify the perceived benefits, barriers and enablers using a post-implementation survey.

Methods

Leveraging funding and policy incentives

Our work has concentrated on making use of the opportunities afforded by existing NHS funding and policy mechanisms to encourage implementation of CRP PoC in primary care. We did not provide any research funding to participating organisations to ensure that successful implementation was not artificial, and could potentially be reproduced by others in the context of the real-world financial structures and constraints associated with healthcare commissioning in the NHS. All work in this area was undertaken during 2015 and 2016.

For peer review only

For peer review only - http://bmjopen.bmj.com/site/about/guidelines.xhtml

132 We developed a standard LES specification to underpin local implementation, establishing a funding framework of reimbursement of general practices by Clinical Commissioning Groups (CCGs) for CRP PoC on a unit basis. In view of the importance given to budgetary concerns by commissioners considering CRP implementation[26], CCGs may be motivated by its potential to open access to national funding associated with achieving the NHS England 'Quality Premium' (QP) target for reduced antibiotic prescribing in primary care[28].

138 Our research group, NIHR CLAHRC Wessex, is funded by both the National Institute for Health Research (NIHR) and partner organisations (including CCGs) within the local health system. Partner funding contributions may be monetary, or comprised of research study involvement. Our locality covers nine CCGs, each of whom had the opportunity to fulfil this funding obligation by participating in a CRP implementation study, or similar research. As well as this benefit, there was further opportunity for any participation costs to be partially or fully offset if the QP was achieved as a result.

145 **Engaging with the NHS**

146 Using materials from the GRACE Intro study[15] we developed resources including an online training course for general practitioners on the use of CRP, a clinical audit form and patient information leaflet[29]. All resources were made available to interested organisations as a means of facilitating implementation by reducing the associated administrative burden.

150 We visited clinicians and healthcare commissioners in our locality to generate interest, and made presentations at locality events to promote the LES framework. We also attended an NIHR CLARHC Wessex showcase event to which local CCGs were invited. We followed up additional enquiries from other groups outside of our locality who were interested in CRP implementation by offering visits and presentations, and sharing the resources developed for our local study. Resources were shared with ten groups across the country.

For peer review only - http://bmjopen.bmj.com/site/about/guidelines.xhtml

156 **Post-implementation survey**

157 In August 2017, following our period of NHS engagement, we issued an electronic survey to a
158 convenience sample of clinicians and commissioners who had expressed an interest in, or were known
159 to have contributed to, CRP implementation projects. Overall, nineteen individuals were invited to
160 participate, including healthcare commissioners, pharmacists, primary and secondary care clinicians,
161 and public health professionals.

162 We adopted a qualitative approach to explore in more depth the factors motivating respondents' initial
163 interest, their experience of the implementation process and perceived barriers and enablers. Survey
164 questions were written in line with these underlying objectives as deductively generated main themes[30]
165 (**box 1**).

We asked participants:

- What were your/your organisation's reasons for implementing CRP testing?
- What was your experience of implementing and using CRP testing, and what is happening now?
- Which aspects of the implementation worked well?
- What were the barriers to implementation and/or continued use?
- How did you overcome these barriers?
- What would have helped, or would help in the future to encourage continued use?
- What would facilitate the implementation process?
- What would be your recommendations for those looking to implement CRP testing in the future?

Box 1: Post-implementation survey questions

166

167 Following the method of thematic analysis described by Nowell and colleagues[31], three members of the
168 research team (MJ, NS, TM) individually reviewed all survey responses to inductively identify more
169 specific subthemes. Reviewers took a systematic and iterative approach to analysis, later using
170 researcher triangulation to reach consensus.

171 **Implementation case study**

172 In parallel with our work to evaluate the use of an item of service payment framework as a means of
173 encouraging CRP implementation, a separate study was undertaken in Herts Valleys CCG to evaluate
174 CRP utilisation over three winter months (November 2016 – January 2017). This case study did not use
175 the LES framework, being separately funded by an NHS England Innovation Challenge Prize and driven

For peer review only - http://bmjopen.bmj.com/site/about/guidelines.xhtml

by a local champion. However, in view of the successful implementation in this locality we present further detail in **Box 2** and results in **Appendix B** to demonstrate the potential effects of implementation of CRP PoC on antibiotic prescribing.

Funded by an NHS England Innovation Challenge Prize, an implementation study was undertaken in Herts Valleys CCG to evaluate CRP utilisation over three winter months (November 2016 – January 2017) and in five general practices, purposively sampled using standardised practice-level prescribing data to target high and medium antibiotic prescribers. The study aimed to evaluate whether, compared to standard care, the availability of CRP PoC for LRTI in primary care was associated with reduced acute and follow-up antibiotic prescribing, and unscheduled primary care reattendances and healthcare contacts in the 28 days following presentation.

Participating practices received an intervention consisting of one testing device and supplies to perform 100 tests, training on the NICE guidelines and equipment use, a review visit and practical assistance from the study team where appropriate; all other costs were borne by the practice. Each practice was free to select an appropriate device location and means of operationalising patient flow based on the physical layout of the practice, available resources and staff skill mix.

In line with the NICE guidelines, patients aged 18-65 presenting to intervention practices with suspected LRTI of less than three weeks' duration where there was diagnostic uncertainty were eligible to receive a test. Eligibility was assessed by the clinician during patient consultation. Patients with acute pneumonia, pregnant, immunocompromised, terminally ill or under follow up for Chronic Obstructive Pulmonary Disease were excluded[21]. As the offer was made on clinician discretion, and the patient entitled to refuse, some eligible patients did not receive a test. However, all eligible patients presenting to intervention practices were included in the evaluation, irrespective of whether they received a test.

The five intervention practices were compared to three Herts Valleys CCG control practices of similar size and prescribing level, all of which continued to provide standard care. Control practices did not receive training. One member of the study team (LC) conducted a retrospective electronic search at control practices to identify new clinical consultations (during the same study period) with patients who met the CRP eligibility criteria. Presentations were identified using a set of Read codes[32] commonly used to record clinical activity related to LRTI in NHS primary care, and relevant information collected for analytical purposes.

Results from the implementation study are given in **Appendix B**.

Box 2: Herts Valleys CCG implementation study

Patient and public involvement

There was no patient and public involvement (PPI) in development of the research question, although implementation of CRP PoC flowed from the NICE pneumonia guidelines[21], the development of which

For peer review only - http://bmjopen.bmj.com/site/about/guidelines.xhtml

involved substantial PPI input. There was no PPI in development of the LES specification. This would not be normal practice in respect of a contractual arrangement for the funding of general practices.

Results

Adoption of the LES framework and implementation of CRP

Whilst there was initial interest in CRP PoC facilitated by use of the LES framework, ultimately no CCGs within the NIHR CLAHRC Wessex locality participated in implementation projects. CRP was under consideration by one local CCG as part of a range of measures that might contribute to achieving a 'Quality, Innovation, Productivity and Prevention' programme target around improving detection of pneumonia in primary care, with the aim of enabling earlier intervention and reducing hospital admissions. The CCG had planned to implement CRP across all of its general practices, but concluded that the associated upfront capital cost was too substantial and did not proceed.

Another CCG outside of our locality was interested in more widespread CRP implementation based on antibiotic prescribing reductions observed during a pilot undertaken in a single general practice. Although ten testing devices were procured and were initially regularly used, declining utilisation in the face of operational barriers prompted the CCG to cease procurement of PoC consumables. Financial incentivisation by way of the LES framework was considered as a means of encouraging utilisation, but ultimately failed to reengage interest.

We are not aware of any other CCGs having adopted the LES framework, or having engaged in implementation projects.

Post-implementation survey

For peer review only - http://bmjopen.bmj.com/site/about/guidelines.xhtml

Of the nineteen individuals invited to participate, seven (37%) submitted full responses. Several subthemes emerged from inductive analysis, with a high level of consistency amongst respondents (**table 1**).

All respondents reported being organisationally motivated by the potential for CRP PoC to help reduce antibiotic prescribing, while some further specified a desire to reduce variation in prescribing rates amongst practices in their locality. However, respondents also described mixed clinician utilisation: while some regularly incorporated PoC into consultations for suspected LRTI, others did not use it at all. Furthermore, one respondent noted that while utilisation had initially been high, it had declined over time.

Benefits:
• A clinical aid to appropriate antibiotic prescribing<br>• An objective measure to improve patient confidence in the prescribing action
Barriers:
• Limited time available during consultation<br>• Layout of facility and placement of testing device<br>• Cost of implementation and continued use<br>• Source of funding<br>• Resistance to change<br>• Maintaining engagement
Enablers:
• Early adopters to share experience and provide mentorship<br>• Training and education<br>• Champions within practice/locality<br>• Collaboration at local and national level<br>• Better utilisation of IT to facilitate testing process
**Table 1: Benefits, barriers and enablers of implementation**

Benefits

Most respondents agreed that CRP is a valuable clinical aid to appropriate antibiotic prescribing for patients with symptoms of LRTI. Furthermore, some highlighted its value as an objective measure to improve patient confidence in the chosen prescribing action, particularly in consultation with those who are "very keen" to receive antibiotics. Two respondents noted that, in their experience, patients had responded positively to the test and were satisfied with the outcome.

For peer review only - http://bmjopen.bmj.com/site/about/guidelines.xhtml

Barriers

In general, respondents reported that interest amongst clinicians was sometimes poor, and suggested a need for financial incentives and support to encourage widespread uptake. Most mentioned cost pressures, while some questioned who should be responsible for funding: general practices or the CCG. Despite the evidence base for the clinical benefits, one respondent suggested that there remains a need to "clearly demonstrate short term benefits in costs, workload and safety" to develop and maintain engagement.

Most respondents commented on the impact of operational constraints, such as the physical layout of the practice, how to accommodate multiple users, and the time required to carry out the test, particularly in the context of high workload and limited consultation duration. Although some respondents argued that other benefits justified its use despite these barriers, others specifically cited them as disincentives, especially for clinicians who may have a negative attitude to CRP or be resistant to change.

Enablers

Most respondents discussed the importance of collaboration, although interpretations of this differed. Some suggested that early adopter sites share lessons learned to help others and avoid duplicated effort. The value of training and education during the implementation process were consistently emphasised, and development of a standard programme was suggested. Others mentioned the role of NIHR in fostering collaborative working, and the potential for general practice or CCG champions to improve engagement and resolve problems. Some respondents also suggested better use of IT to facilitate testing. Specific examples included the deployment of standard templates to record the test and result in the practice management system, and use of electronic alerts during consultation to prompt clinicians to PoC if indicated.

For peer review only

For peer review only - http://bmjopen.bmj.com/site/about/guidelines.xhtml

Discussion

Summary of main findings

Despite initial interest, there was no implementation in the NIHR CLAHRC Wessex locality, and no CCGs formally adopted the LES framework. The research team were unable to gain significant traction with CCG management, and when contact was established CCGs were unwilling to prioritise antibiotic stewardship over other local initiatives. The policy levers seemed to have little impact in this locality, where CCGs were struggling to remain in budget. The financial rewards arising from the QP only applied to CCGs meeting financial targets. Elsewhere, one CCG implemented CRP and, following declining utilisation in response to operational barriers, found that the LES framework was insufficient as a mechanism to reengage interest.

Although the small sample size limits inference and generalisability, our post-implementation survey identified several financial, operational and physical barriers in common with previous qualitative research[26]. Respondents confirmed that implementation would be unlikely without financial incentives but also highlighted difficulties integrating PoC into practice workflow, and constraints arising from a lack of dedicated space, equipment sharing and limited time. Reported enablers included adequate training and the value of a local champion.

Some respondents also emphasised the clinical benefits of CRP, giving anecdotal examples of cases where testing had prevented antibiotic prescription. The potential for more widespread repetition of this outcome is suggested by quantitative results from the Herts Valley CCG implementation study, where a successful, separately funded implementation scheme was run for a three month period, driven by a local champion. Observation of substantial prescribing reductions amongst intervention practices suggests that implementation in the NHS might replicate the prescribing reductions reported in research studies.

Comparison with other literature

For peer review only - http://bmjopen.bmj.com/site/about/guidelines.xhtml

We are unaware of any other implementation studies concerning CRP PoC in the UK. In other health settings PoC is widely adopted[22], and following government directives has been introduced in the Netherlands[33]. The financial barriers to implementation have been identified in a previous study including European and UK participants[23], which noted that countries with high rates of use had alternative reimbursement models, and that widespread implementation in Europe followed health policy change. The same study also highlighted issues around workflow and time as potential barriers to implementation in the UK.

Strengths and weaknesses

Our study describes the results of attempts at CRP implementation without the resources associated with research, and without specific policy directives. It is unclear how generalisable our findings might be; it would appear that CCG partnership with NIHR CLAHRC Wessex and national level incentives via the QP should have maximised the potential for local implementation. The scheme was devised during a time of general financial constraint within the NHS, which may have had particular impact in the Wessex locality.

Limitations around funding mechanisms

The criteria required to achieve the QP, even taking the antibiotic prescribing element alone, has been inconsistent. Some changes have been significant, such as a move to greater emphasis on antibiotic prescribing for urinary tract infection as of 2018/19[28]. Furthermore, as the QP is awarded retrospectively and is contingent upon meeting other financial targets, the funding mechanism is not guaranteed, making it difficult to engage commissioners and to create a firm financial framework to underpin CRP implementation.

A further feature of the QP is that no method of achievement is stipulated; the antibiotic prescribing element simply requires an absolute prescribing reduction. The NHS has reported a national ~7% reduction in primary care coinciding with the implementation phase of this study[34-36], which may

For peer review only - http://bmjopen.bmj.com/site/about/guidelines.xhtml

have resulted from a general policy shift and increased focus of clinical training in primary care. This suggests that overall improvements could be gained and the QP target potentially achieved by way of alternative, lower cost methods alone, negating commissioners' financial incentive for CRP implementation irrespective of the clinical benefits.

The pressures of multiple, competing commissioning programmes may limit engagement with certain initiatives, while the overall funding structure of the NHS may also influence commissioners' preferences and priorities. One CCG within our locality suggested that, despite evidence of a net cost saving associated with CRP[37], whilst the upfront implementation costs reside with primary care, any savings would principally be realised by the secondary care sector. In this instance, therefore, concerns that the costs and benefits of the initiative may be distinctly localised within separate areas of the health system acted as a disincentive to its adoption.

Implications

Whilst the use of existing financial structures appeared appealing as a mechanism, it was not possible to fully test the hypothesis that modest financial incentives to general practices at local level would enable CRP implementation, as financial pressures impeded CCG adoption of the policy. National incentives for CCGs did not appear to override the financial constraints because a) financial rewards were only available to CCGs meeting financial targets, and b) antibiotic targets were being achieved through other mechanisms not requiring financial investment.

Although a small case study suggests that implementation outside of research studies may result in similar prescribing reductions, since it was driven by local investment and a local champion it may not fully reflect implementation in routine practice, or be generalisable to other areas. Furthermore, and recalling questions over the primacy of lower cost measures, the fact that this intervention provided training and support in addition to testing materials limits the extent to which the observed prescribing reductions can be confidently attributed to CRP PoC alone.

For peer review only - http://bmjopen.bmj.com/site/about/guidelines.xhtml

317 The value of an enthusiastic, local champion to catalyse support for implementation emerged from
318 both the qualitative and quantitative strands of this study. Knowledge mobilisation and
319 implementation in practice may be assisted by way of a Researcher-In-Residence model[38], while
320 further qualitative and observational research could improve understanding of how champions are
321 able to persuade and engage clinicians and to encourage commissioners to look beyond the
322 immediate financial disincentives, and whether they may be effective in other areas and settings.
323 Further economic research might also model different modes of implementation to assess the costs
324 and consequences across the system, and to find alternative funding models to overcome the
325 financial barriers. Multi-purpose testing devices, for example, may have the advantage of spreading
326 investment across several funding streams.

327 In conclusion, it seems unlikely that financial schemes falling outside of national policy will gain
328 much traction in a financially constrained NHS. Full-scale implementation of CRP PoC is likely to
329 require central implementation via government policy or contractual changes.

330

331 List of abbreviations

332 LRTI: Lower respiratory tract infection; NPT: Near patient test; CRP: C-reactive protein; PCT:
333 Procalcitonin; NICE: National Institute for Clinical Excellence; NHS: National Health Service; PoC:
334 Point of care testing; LES: Locally Enhanced Service; CCG: Clinical Commissioning Group; QP: NHS
335 England Quality Premium; NIHR: National Institute for Health Research; PPI: Patient and public
336 involvement

337

338 Declarations

339 Ethical approval

340 The Integrated Research Application System (IRAS) confirmed that formal ethical approval was not
341 required for the Herts Valleys CCG implementation study; a service evaluation project.

For peer review only - http://bmjopen.bmj.com/site/about/guidelines.xhtml

Funding statement

This research was funded by the National Institute for Health Research (NIHR) Collaboration for Leadership in Applied Health Research and Care Wessex at University Hospital Southampton NHS Foundation Trust. The views expressed are those of the author(s) and not necessarily those of the NHS, the NIHR or the Department of Health and Social Care.

The Herts Valleys CCG implementation study was separately supported by a 2015/16 NHS England Innovation Challenge Prize, Acorn award.

Competing interests

MJ, NS, JS, TM and MM have no competing interests to declare. LC has received honoraria from Abbott Laboratories and Roche Diagnostics Ltd for speaking events.

Authors' contributions

MJ carried out quantitative analysis and wrote the paper with contributions from MM. MJ and NS developed the post-implementation survey and, with TM, carried out qualitative analysis. LC carried out the Herts Valleys CCG implementation study. JS, NS and MM developed the LES framework and other resources, and engaged with the NHS. TM provided methodological input and MM provided clinical guidance. All authors commented on drafts of the paper and have read and approved the final manuscript.

Patient consent for publication

Not applicable.

Data sharing statement

No additional data available.

Acknowledgements

The authors thank Elsie Griffins and Dr Denise Knight for their support for the Herts Valleys CCG implementation study.

For peer review only - http://bmjopen.bmj.com/site/about/guidelines.xhtml

References

1. Butler CC, Hood K, Verheij T, et al. Variation in antibiotic prescribing and its impact on recovery in patients with acute cough in primary care: prospective study in 13 countries. BMJ 2009;338(b2242)
2. Akkerman AE, van der Wouden JC, Kuyvenhoven MM, et al. Antibiotic prescribing for respiratory tract infections in Dutch primary care in relation to patient age and clinical entities. *J Antimicrob Chemother* 2004;54(6):1116-21. doi: 10.1093/jac/dkh480 [published Online First: 2004/11/18]
3. Davies SC. Annual Report of the Chief Medical Officer, Volume Two, 2011, Infections and the rise of antimicrobial resistance. London: Department of Health 2013.
4. Smith SM, Fahey T, Smucny J, et al. Antibiotics for acute bronchitis. *Cochrane Database Syst Rev* 2014(3):CD000245. doi: 10.1002/14651858.CD000245.pub3 [published Online First: 2014/03/04]
5. Little P, Stuart B, Moore M, et al. Amoxicillin for acute lower-respiratory-tract infection in primary care when pneumonia is not suspected: a 12-country, randomised, placebo-controlled trial. *The Lancet Infectious Diseases* 2013;13(2):123-29. doi: 10.1016/s1473-3099(12)70300-6
6. Moore M, Stuart B, Coenen S, et al. Amoxicillin for acute lower respiratory tract infection in primary care: subgroup analysis of potential high-risk groups. *Br J Gen Pract* 2014;64(619):e75-80. doi: 10.3399/bjgp14X677121 [published Online First: 2014/02/26]
7. Cornford CS. Why patients consult when they cough: a comparison of consulting and non-consulting patients. *British Journal of General Practice* 1998;48(436):1751-4.
8. Little P, Watson L, Morgan S, et al. Antibiotic prescribing and admissions with major suppurative complications of respiratory tract infections: a data linkage study. British Journal of General Practice 2002;52:187-93.9. Winchester CC, Macfarlane TV, Thomas M, et al. Antibiotic prescribing and outcomes of lower respiratory tract infection in UK primary care. *Chest* 2009;135(5):1163-72. doi: 10.1378/chest.07-2940 [published Online First: 2009/05/08]
10. Moore M, Little P, Rumsby K, et al. Effect of antibiotic prescribing strategies and an information leaflet on longer-term reconsultation for acute lower respiratory tract infection. *Br J Gen Pract* 2009;59(567):728-34. doi: 10.3399/bjgp09X472601 [published Online First: 2009/10/22]
11. Goossens H, Ferech M, Vander Stichele R, et al. Outpatient antibiotic use in Europe and association with resistance: a cross-national database study. *The Lancet* 2005;365(9459):579-87. doi: 10.1016/s0140-6736(05)70799-6
12. Little P, Stuart B, Smith S, et al. Antibiotic prescription strategies and adverse outcome for uncomplicated lower respiratory tract infections: prospective cough complication cohort (3C) study. *BMJ* 2017;357:j2148. doi: 10.1136/bmj.j2148 [published Online First: 2017/05/24]
13. Cals JW, Butler CC, Hopstaken RM, et al. Effect of point of care testing for C reactive protein and training in communication skills on antibiotic use in lower respiratory tract infections: cluster randomised trial. *BMJ* 2009;338:b1374. doi: 10.1136/bmj.b1374 [published Online First: 2009/05/07]
14. Cals JW, Schot MJ, de Jong SA, et al. Point-of-care C-reactive protein testing and antibiotic prescribing for respiratory tract infections: a randomized controlled trial. *Ann Fam Med* 2010;8(2):124-33. doi: 10.1370/afm.1090 [published Online First: 2010/03/10]
15. Little P, Stuart B, Francis N, et al. Effects of internet-based training on antibiotic prescribing rates for acute respiratory-tract infections: a multinational, cluster, randomised, factorial, controlled trial. *The Lancet* 2013;382(9899):1175-82. doi: 10.1016/s0140-6736(13)60994-0
16. Briel M, Schuetz P, Mueller B, et al. Procalcitonin-Guided Antibiotic Use vs a Standard Approach for Acute Respiratory Tract Infections in Primary Care. Arch Intern Med 2008;168(18):2000-07.

For peer review only - http://bmjopen.bmj.com/site/about/guidelines.xhtml

17. Holm A, Pedersen SS, Nexoe J, et al. Procalcitonin versus C-reactive protein for predicting pneumonia in adults with lower respiratory tract infection in primary care. British Journal of General Practice 2007;57:555-60.
18. Christ-Crain M, Opal SM. Clinical review: The role of biomarkers in the diagnosis and management of community acquired pneumonia. *Critical Care* 2010;14(203) doi: 10.1186/cc8155
19. Schuetz P, Briel M, Christ-Crain M, et al. Procalcitonin to guide initiation and duration of antibiotic treatment in acute respiratory infections: an individual patient data meta-analysis. *Clin Infect Dis* 2012;55(5):651-62. doi: 10.1093/cid/cis464 [published Online First: 2012/05/11]
20. Aabenhus R, Jensen JU, Jorgensen KJ, et al. Biomarkers as point-of-care tests to guide prescription of antibiotics in patients with acute respiratory infections in primary care. *Cochrane Database Syst Rev* 2014(11):CD010130. doi: 10.1002/14651858.CD010130.pub2 [published Online First: 2014/11/07]
21. National Institute for Health and Care Excellence. Pneumonia in adults: diagnosis and management (CG191) 2014 [Available from: https://www.nice.org.uk/guidance/cg191 accessed 13 March 2018.
22. André M, Schwan A, Odenholt I. The Use of CRP Tests in Patients with Respiratory Tract Infections in Primary Care in Sweden Can Be Questioned. *Scandinavian Journal of Infectious Diseases* 2009;36(3):192-97. doi: 10.1080/00365540410019372
23. Minnaard MC, van de Pol AC, Hopstaken RM, et al. C-reactive protein point-of-care testing and associated antibiotic prescribing. *Fam Pract* 2016;33(4):408-13. doi: 10.1093/fampra/cmw039 [published Online First: 2016/05/28]
24. Jakobsen KA, Melbye H, Kelly MJ, et al. Influence of CRP testing and clinical findings on antibiotic prescribing in adults presenting with acute cough in primary care. *Scand J Prim Health Care* 2010;28(4):229-36. doi: 10.3109/02813432.2010.506995 [published Online First: 2010/08/14]
25. Tonkin-Crine S, Yardley L, Coenen S, et al. GPs' views in five European countries of interventions to promote prudent antibiotic use. *Br J Gen Pract* 2011;61(586):e252-61. doi: 10.3399/bjgp11X572445 [published Online First: 2011/05/31]
26. Huddy JR, Ni MZ, Barlow J, et al. Point-of-care C reactive protein for the diagnosis of lower respiratory tract infection in NHS primary care: a qualitative study of barriers and facilitators to adoption. *BMJ Open* 2016;6(3):e009959. doi: 10.1136/bmjopen-2015-009959 [published Online First: 2016/03/05]
27. NHS England. NHS England Standard General Medical Services Contract 2017/18 2018 [Available from: https://www.england.nhs.uk/gp/gpfv/investment/gp-contract/ accessed 13 March 2018.
28. NHS England. Quality Premium 2018 [Available from: https://www.england.nhs.uk/resources/resources-for-ccgs/ccg-out-tool/ccg-ois/qual-prem/ accessed 16 May 2018.
29. NIHR CLAHRC Wessex. Reducing unnecessary antibiotic prescribing in primary care 2018 [Available from: https://www.clahrc-wessex.nihr.ac.uk/theme/project/20 accessed 24th May 2018.
30. Braun V, Clarke V. Using thematic analysis in psychology. *Qualitative Research in Psychology* 2006;3(2):77-101. doi: 10.1191/1478088706qp063oa
31. Nowell LS, Norris JM, White DE, et al. Thematic Analysis: Striving to Meet the Trustworthiness Criteria. *International Journal of Qualitative Methods* 2017;16:1-13. doi: 10.1177/1609406917733847
32. Benson T. The history of the Read codes: the inaugural James Read Memorial Lecture 2011. *Journal of Innovation in Health Informatics* 2011;19(3):173-82. doi: 10.14236/jhi.v19i3.811

For peer review only - http://bmjopen.bmj.com/site/about/guidelines.xhtml

33. The Dutch College of General Practitioners (NHG). NHG-Standaard Acuut hoesten (Acute cough guidelines) 2018 [Available from: https://www.nhg.org/standaarden/volledig/nhg-standaard-acuut-hoesten accessed 31st May 2018.
34. NHS England. Success in NHS push to reduce avoidable antibiotic prescribing 2016 [Available from: https://www.england.nhs.uk/2016/03/antibiotic-prescribing/ accessed 17 May 2018.
35. NHS England. Antibiotic quality premium monitoring dashboard 2018 [Available from: https://www.england.nhs.uk/resources/resources-for-ccgs/ccg-out-tool/ccg-ois/anti-dash/ accessed 17 May 2018.
36. NHS Improvement. Helping GPs to cut antibiotic prescriptions by 2.6m in just one year 2016 [Available from: https://improvement.nhs.uk/news-alerts/helping-gps-cut-antibiotic-prescriptions/ accessed 17 May 2018.
37. Hunter R. Cost-effectiveness of point-of-care C-reactive protein tests for respiratory tract infection in primary care in England. *Adv Ther* 2015;32(1):69-85. doi: 10.1007/s12325-015-0180-x [published Online First: 2015/01/27]
38. Marshall M, Pagel C, French C, et al. Moving improvement research closer to practice: the Researcher-in-Residence model. *BMJ Qual Saf* 2014;23(10):801-5. doi: 10.1136/bmjqs-2013-002779 [published Online First: 2014/06/05]

For peer review only - http://bmjopen.bmj.com/site/about/guidelines.xhtml

Appendix A: Primary care testing in the NHS health system

In the UK healthcare is free at the point of contact and funded through central taxation. There is no co-payment or health insurance needed for patients to access the NHS. General practices rarely perform tests on the primary care site, with few exceptions such as urine dip tests, pregnancy tests and sometimes coagulation monitoring. The majority of laboratory tests are organised by the local hospital, with patients either attending the hospital directly for sampling, or samples being taken at the general practice and sent to the laboratory. Payment for the test is by way of a 'block contract' arrangement, and paid for by the CCG so that neither the patient nor the practice bear any cost. Under the current contractual arrangement the general practice is unable to charge the patient for NPTs and so, if performed, bears the full cost from its own income, as well as an additional time burden. For these reasons NPTs such as CRP are unfamiliar to practitioners in the UK, necessitating an examination of alternative means to encourage their implementation.

For peer review only

Appendix B: Results from Herts Valleys CCG implementation study

Five intervention practices with a total list size of 63,743 patients recorded 682 eligible LRTI presentations during the study period, of which 176 (26%) involved a CRP test. Three control practices recorded 258 presentations (based on the same eligibility criteria) from 35,928 patients.

The conversion of initial LRTI presentations to CRP tests (intervention arm only) and to other primary care healthcare events (both study arms) were reported descriptively. A binary outcome variable was created to represent antibiotic prescription during the 28 days following the initial LRTI presentation. As delayed prescribing was relatively infrequent at both intervention and control practices acute and delayed prescriptions were combined into a single outcome. Multivariate logistic regression was then used to estimate the odds of antibiotic prescription and follow-up consultation following initial presentation to practices in the intervention and control arms, adjusting for age (modelled as a binary variable with categories '< 44' and '≥ 45') and sex. Model fit was assessed using the likelihood ratio test, which indicated that patient sex was not a statistically significant predictor of either outcome. Adjusted odds ratios from the final models were reported, along with 95% confidence intervals and p-values to assess significance.

Overall, fewer initial presentations to intervention practices resulted in antibiotic prescription (59% of initial presentations, as compared to 79%) and follow-up consultations (30% compared to 38%), although there was little difference to antibiotic prescribing at follow-up (both arms 68%) (**table 2**). Furthermore, initial presentations with antibiotic prescription then resulting in follow-up consultation with an additional prescription were more common amongst control practices (21% compared to 13%).

For peer review only

	Intervention arm (n = 682)		Control arm (n = 258)	
	Outcome events	%	Outcome events	%
**CRP test at initial presentation**	176	26	-	-
**Antibiotic prescription at initial presentation**	405	59	204	79
**Follow-up consultation after initial presentation**	206	30	99	38
**Antibiotic prescription at follow-up consultation**	140	68 [a]	67	68 [a]
**Initial presentation with antibiotic prescription, then follow-up consultation with additional antibiotic prescription**	92	13	55	21

Table 2: Primary care healthcare events resulting from initial LRTI presentation
All percentages compared to number of initial presentations, except ([a]) compared to number of follow up consultations

We found that the odds of antibiotic prescribing after initial presentation were reduced by 62% amongst intervention practices, and the odds of follow up consultation were reduced by 32% (**table 3**). In each case we found that the outcome was more likely amongst presenting patients in the older age category.

Outcome	Variable	Adjusted OR (95% CI)	p-value
**Antibiotic prescription after initial presentation**	**Study arm**		
	Control	*Reference*	< 0.001
	Intervention	0.38 (0.27, 0.53)	
	**Patient age**		
	< 44	*Reference*	0.035
	≥ 45	1.35 (1.02, 1.77)	
**Follow-up consultation after initial presentation**	**Study arm**		
	Control	*Reference*	0.013
	Intervention	0.68 (0.51, 0.92)	
	**Patient age**		
	< 44	*Reference*	0.019
	≥ 45	1.40 (1.06, 1.85)	

Table 3: Multivariate logistic regression models for the association of practice-level intervention with antibiotic prescribing after initial presentation and follow-up consultation after initial presentation

For peer review only

STROBE 2007 (v4) checklist of items to be included in reports of observational studies in epidemiology*
Checklist for cohort, case-control, and cross-sectional studies (combined)

Section/Topic	Item #	Recommendation	Reported on page #
**Title and abstract**	1	(*a*) Indicate the study's design with a commonly used term in the title or the abstract	Mixed-methods design reported in title (page 1), and described in more detail in abstract (page 2)
		(*b*) Provide in the abstract an informative and balanced summary of what was done and what was found	Page 2
**Introduction**			
Background/rationale	2	Explain the scientific background and rationale for the investigation being reported	Page 3-5
Objectives	3	State specific objectives, including any pre-specified hypotheses	Page 5
**Methods**			
Study design	4	Present key elements of study design early in the paper	Described briefly in abstract (page 2), and in more detail in methods (page 5-8)
Setting	5	Describe the setting, locations, and relevant dates, including periods of recruitment, exposure, follow-up, and data collection	Separately reported for engagement work (page 5-6), qualitative component (page 7), and quantitative component (page 8)
Participants	6	(*a*) *Cohort study*—Give the eligibility criteria, and the sources and methods of selection of participants. Describe methods of follow-up<br>*Case-control study*—Give the eligibility criteria, and the sources and methods of case ascertainment and control selection. Give the rationale for the choice of cases and controls<br>*Cross-sectional study*—Give the eligibility criteria, and the sources and methods of selection of participants	Separately reported for engagement work (page 5-6), qualitative component (page 7), and quantitative component (page 8)
		(*b*) *Cohort study*—For matched studies, give matching criteria and number of exposed and unexposed<br>*Case-control study*—For matched studies, give matching criteria and the number of controls per case	Not applicable. Quantitative component used intervention/control arms, but not matched; purposive sample and basis for comparison described (page 8)
Variables	7	Clearly define all outcomes, exposures, predictors, potential confounders, and effect modifiers. Give diagnostic criteria, if applicable	Described for quantitative component (Appendix B)
Data sources/ measurement	8*	For each variable of interest, give sources of data and details of methods of assessment (measurement).	Described for quantitative

For peer review only

		Describe comparability of assessment methods if there is more than one group	component (page 8)
Bias	9	Describe any efforts to address potential sources of bias	Acknowledged potential bias arising from quantitative component (page 14), however not possible to quantify and address in analysis
Study size	10	Explain how the study size was arrived at	Recruitment of participants/study size separately reported for engagement work (page 6), qualitative component (page 7), and quantitative component (page 8)
Quantitative variables	11	Explain how quantitative variables were handled in the analyses. If applicable, describe which groupings were chosen and why	Reported for quantitative component (Appendix B)
Statistical methods	12	(*a*) Describe all statistical methods, including those used to control for confounding	Reported for quantitative component (Appendix B)
		(*b*) Describe any methods used to examine subgroups and interactions	Not applicable
		(*c*) Explain how missing data were addressed	Not applicable
		(*d*) *Cohort study*—If applicable, explain how loss to follow-up was addressed<br>*Case-control study*—If applicable, explain how matching of cases and controls was addressed<br>*Cross-sectional study*—If applicable, describe analytical methods taking account of sampling strategy	Not applicable
		(*e*) Describe any sensitivity analyses	Not applicable
**Results**			
Participants	13*	(a) Report numbers of individuals at each stage of study—eg numbers potentially eligible, examined for eligibility, confirmed eligible, included in the study, completing follow-up, and analysed	Reported for quantitative component (Appendix B), including eligibility criteria (page 8)
		(b) Give reasons for non-participation at each stage	Not applicable
		(c) Consider use of a flow diagram	Not applicable
Descriptive data	14*	(a) Give characteristics of study participants (eg demographic, clinical, social) and information on exposures and potential confounders	Not applicable
		(b) Indicate number of participants with missing data for each variable of interest	Not applicable
		(c) *Cohort study*—Summarise follow-up time (eg, average and total amount)	Reported duration of quantitative component follow-up period (page 8 and Appendix B)

For peer review only

Outcome data	15*	*Cohort study*—Report numbers of outcome events or summary measures over time	Reported for quantitative component (Appendix B)
		Case-control study—Report numbers in each exposure category, or summary measures of exposure	Not applicable
		Cross-sectional study—Report numbers of outcome events or summary measures	Not applicable
Main results	16	(*a*) Give unadjusted estimates and, if applicable, confounder-adjusted estimates and their precision (eg, 95% confidence interval). Make clear which confounders were adjusted for and why they were included	Quantitative analysis reported briefly in appendix (Appendix B). Adjusted odds ratios presented, with confidence intervals and confounding variables
		(*b*) Report category boundaries when continuous variables were categorized	Reported for quantitative component (Appendix B)
		(*c*) If relevant, consider translating estimates of relative risk into absolute risk for a meaningful time period	Not applicable
Other analyses	17	Report other analyses done—eg analyses of subgroups and interactions, and sensitivity analyses	Not applicable
**Discussion**			
Key results	18	Summarise key results with reference to study objectives	Reported for all study components throughout discussion (page 11-15)
Limitations	19	Discuss limitations of the study, taking into account sources of potential bias or imprecision. Discuss both direction and magnitude of any potential bias	Overall study limitations discussed (page 13), including specific acknowledgement of potential for bias arising from quantitative component (page 14)
Interpretation	20	Give a cautious overall interpretation of results considering objectives, limitations, multiplicity of analyses, results from similar studies, and other relevant evidence	Overall interpretation of results discussed (page 13-14)
Generalisability	21	Discuss the generalisability (external validity) of the study results	Statements on generalisability given (page 12, 14)
**Other information**			
Funding	22	Give the source of funding and the role of the funders for the present study and, if applicable, for the original study on which the present article is based	Page 15

*Give information separately for cases and controls in case-control studies and, if applicable, for exposed and unexposed groups in cohort and cross-sectional studies.

For peer review only

Note: An Explanation and Elaboration article discusses each checklist item and gives methodological background and published examples of transparent reporting. The STROBE checklist is best used in conjunction with this article (freely available on the Web sites of PLoS Medicine at http://www.plosmedicine.org/, Annals of Internal Medicine at http://www.annals.org/, and Epidemiology at http://www.epidem.com/). Information on the STROBE Initiative is available at www.strobe-statement.org.

For peer review only
